# HaCaT Cells as a Reliable In Vitro Differentiation Model to Dissect the Inflammatory/Repair Response of Human Keratinocytes

**DOI:** 10.1155/2017/7435621

**Published:** 2017-12-17

**Authors:** Irma Colombo, Enrico Sangiovanni, Roberta Maggio, Carlo Mattozzi, Stefania Zava, Yolanda Corbett, Marco Fumagalli, Claudia Carlino, Paola Antonia Corsetto, Diletta Scaccabarozzi, Stefano Calvieri, Angela Gismondi, Donatella Taramelli, Mario Dell'Agli

**Affiliations:** ^1^Dipartimento di Scienze Farmacologiche e Biomolecolari, Università degli Studi di Milano, Via Balzaretti 9, 20133 Milano, Italy; ^2^Dipartimento di Medicina Sperimentale, Università di Roma La Sapienza, Viale Regina Elena 324, 00161 Roma, Italy; ^3^Dipartimento di Medicina Interna e Specialità Mediche, UOC di Clinica Dermatologica, Università di Roma “Sapienza”, Viale del Policlinico 155, 00161 Roma, Italy; ^4^Center for Life Nano Science@Sapienza, Istituto Italiano di Tecnologia, Viale Regina Elena 291, 00161 Roma, Italy; ^5^Dipartimento di Medicina Molecolare, Università di Roma La Sapienza, Viale Regina Elena 291, 00161 Roma, Italy

## Abstract

Cultured primary human keratinocytes are frequently employed for studies of immunological and inflammatory responses; however, interpretation of experimental data may be complicated by donor to donor variability, the relatively short culture lifetime, and variations between passages. To standardize the *in vitro* studies on keratinocytes, we investigated the use of HaCaT cells, a long-lived, spontaneously immortalized human keratinocyte line which is able to differentiate *in vitro*, as a suitable model to follow the release of inflammatory and repair mediators in response to TNF*α* or IL-1*β*. Different treatment conditions (presence or absence of serum) and differentiation stimuli (increase in cell density as a function of time in culture and elevation of extracellular calcium) were considered. ELISA and Multiplex measurement technologies were used to monitor the production of cytokines and chemokines. Taken together, the results highlight that Ca^2+^ concentration in the medium, cell density, and presence of serum influences at different levels the release of proinflammatory mediators by HaCaT cells. Moreover, HaCaT cells maintained in low Ca^2+^ medium and 80% confluent are similar to normal keratinocytes in terms of cytokine production suggesting that HaCaT cells may be a useful model to investigate anti-inflammatory interventions/therapies on skin diseases.

## 1. Introduction

The skin is a continuously self-renewing organ that dynamically manages the outside-inside-outside relationships of the human body and actively participates in the host defenses [1]. Keratinocytes (KCs) represent 95% of the epidermal cells. Primarily, they play the structural and barrier function of the epidermis, but their role in the initiation and perpetuation of skin inflammatory and immunological responses, and wound repair, is also well recognized [2].

Under homeostatic conditions, KCs differentiate and mature from proliferating nucleated basal cells to the highly differentiated, nucleus-free corneocytes. Each stage of differentiation is characterized by the expression of structural proteins, such as keratins (K) and lipids [3, 4]. For example, the expression of K5 and K14 is restricted to the basal layer, whereas K1 and K10 appear on more differentiated suprabasal cells [4], and involucrin, loricrin, and keratolinin, in the cells of the uppermost spinous layer [1]. Terminal differentiation is driven by various cytokines and growth factors, and it is typically associated with the formation of the peripheral envelopes, rich in proteins and lipids [3, 4]. The calcium (Ca^2+^) gradient in the epidermis, increasing from the basal to the granular layer, represents one of the most important triggers of KC differentiation [4].

Resting KCs produce epidermal growth factor receptor (EGFR) ligands and vascular endothelial growth factor (VEGF), but when activated by bacterial products or by direct damage by UV light or chemicals, the expression of cytokines and chemokines changes [5, 6]. In skin diseases, such as psoriasis [7] or atopic dermatitis [8], the cytokine/chemokine network is even more complex and autocrine/paracrine loops are described [2].

Cultured human KCs are frequently employed for studies of KC functions in chronic inflammatory skin diseases [9, 10]. Primary KCs cultured *in vitro* at low Ca^2+^ concentration retain a basal phenotype, and they differentiate upon addition of Ca^2+^ > 0.1 mM [11, 12]. However, their use for routine monitoring of the inflammatory response of the inflamed skin presents major drawbacks. Firstly, fresh human KCs require supplementary growth factors to survive and proliferate *in vitro*; secondly, once induced to differentiate, they rapidly die and do not allow long-term investigation of the differentiation signals [13]. Moreover, donor-to-donor variability in growth characteristics and *in vitro* responses, different plating efficiencies, the short lifetime in culture, and the changes in proliferation and differentiation characteristics with increasing number of passages, complicates the interpretation of experimental data.

To minimize these problems, the spontaneously immortalized human KC cell line HaCaT from adult skin has been proposed as a model for the study of KC functions. HaCaT is a nontumorigenic monoclonal cell line, adapted to long-term growth without feed-layer or supplemented growth factors [13, 14]; it exhibits normal morphogenesis and expresses all the major surface markers and functional activities of isolated KC [14]; upon stimulation, HaCaT cells differentiate and express specific markers of differentiation, such as K14, K10, and involucrin. They can also form stratified epidermal structure [15], but they can revert, back and forth, between a differentiated and a basal state upon changes in Ca^2+^ concentration in the medium [16]; they retain the capacity to reconstitute a well-structured epidermis after transplantation *in vivo* [17].

The aim of the present study was to investigate and optimize the best conditions to use HaCaT cells as a reliable *in vitro* model to evaluate, at different stages of differentiation, the production of proinflammatory mediators, chosen among those mostly involved in skin inflammation and angiogenesis.

## 2. Materials and Methods

### 2.1. Cell Culture

**Figure 1:**
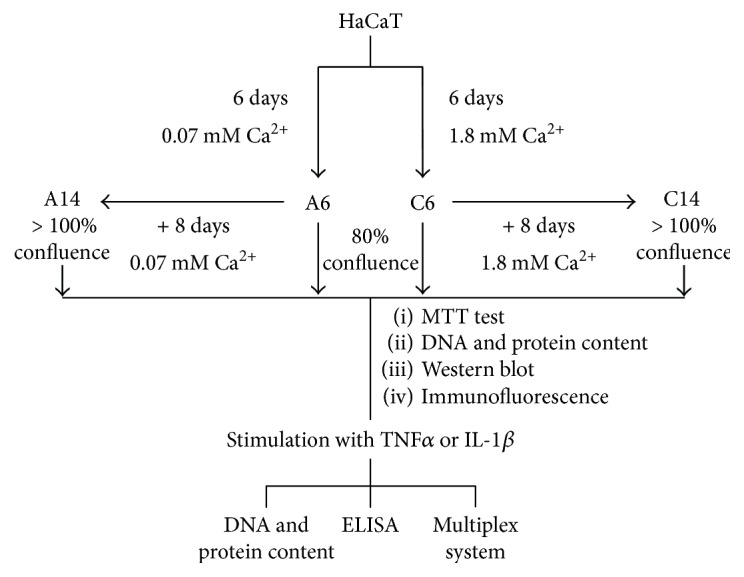
A flow chart with details of the experimental protocol performed on HaCaT cells.

HaCaT cells, spontaneously immortalized human keratinocyte line [15], were kindly provided by Cell Line Service GmbH (Eppelheim, Germany) and cultured in 5% CO_2_ at 37°C in regular Dulbecco's Modified Eagle's Medium (DMEM) (Euroclone S.P.A., Milan, Italy) containing 1.8 mM Ca^2+^, or with DMEM (Gibco, Life Technologies, Carlsbad, CA, USA) at low concentration of Ca^2+^ (0.07 mM). Both media were supplemented with 10% heat-inactivated fetal bovine serum, glutamine (2 mM), penicillin (100 U⁄ml) (Euroclone), and streptomycin (100 mg⁄ml) (Euroclone). For all experiments, cells were seeded at a density of 5.7 × 10^3^ cells⁄cm^2^ and cultured with DMEM at high or low Ca^2+^concentration for 6 or 14 days. The samples were labeled as follows: A6, cells cultured for 6 days with low Ca^2+^ concentration (0.07 mM) and tested when 80% confluent; A14, cells cultured for 14 days with low Ca^2+^ concentration (0.07 mM) and tested when overconfluent; C6, cells cultured for 6 days with high Ca^2+^ concentration (1.8 mM) and tested when 80% confluent; and C14, cells cultured for 14 days with high Ca^2+^ concentration (1.8 mM) and tested when overconfluent. The medium was changed every 2 days. A flow chart with details of the experimental protocol is reported in Figure 1.

### 2.2. Isolation of Human Keratinocytes from Skin Biopsies

Primary KCs were isolated from nonlesional skin biopsies obtained from adult psoriatic patients not receiving either topical or systemic therapies for at least 6 months, or at the time of sample collection. To separate the epidermal layer from the basement membrane, the 0.4 mm punch biopsy was treated with dispase (Gibco BRL, Gaithersburg, MD, USA). After 18 h at 4°C, the epidermal sheet was separated mechanically and dissociated with TrypLE (Gibco BRL, Gaithersburg, MD, USA) for 20 min at 37°C. The obtained primary cells were then plated on 6-well tissue culture plates (Costar), precoated with coating matrix (type I collagen, Gibco BRL), cultured using a specific keratinocyte-serum-free media at low Ca^2+^ concentration (<0.07 mM), and supplemented with human keratinocyte growth factors (Gibco BRL). When the monolayer reached 60%–70% confluence, cells were split by trypsinization. For all the experiments, keratinocyte cultures between the third and fourth passages were used. Informed consent was obtained from all donors providing tissue samples, and ethical approval was obtained from the Ethics Committee of “La Sapienza” University, Rome, Italy.

### 2.3. Cell Proliferation Assay

The proliferation of HaCaT cells was determined at the indicated intervals using the MTT colorimetric assay as described [18]. This test is based on the ability of succinic dehydrogenase of living cells to reduce the yellow salt MTT (3-(4,5-dimethylthiazol-2-yl-2,5-diphenyltetrazolium bromide)) (Sigma-Aldrich, St. Louis, MO, USA) to a purple-blue insoluble formazan precipitate. Experiments were performed in 96-well plates containing a final volume of 100 *μ*l of medium/well. Cells were seeded at an initial density of 1.0 × 10^4^ cells/cm^2^, and, after 1, 6, 9, and 14 days, incubation medium was removed and replaced by 100 *μ*l of fresh medium. Then, 10 *μ*l of stock MTT solution (5 mg/ml in PBS) was added and plates were incubated at 37°C for 4 h. Finally, 100 *μ*l of 10% sodium dodecyl sulfate (SDS) (Sigma-Aldrich, St. Louis, MO, USA), in 0.01 M HCl, was added to each well and the amount of formazan formed was measured at 540 nm using a Benchmark microplate reader (Bio-Rad, Richmond, CA, USA). Sixteen wells per time-point were analyzed in three independent experiments.

### 2.4. Cell Stimulation

For the stimulation of HaCaT cells, an amount of 1.0 × 10^4^ cells/well in 500 *μ*l were seeded in 24-well plate and cultured in low or high Ca^2+^ medium, with medium changes every two days. After 6 or 14 days, the medium was removed and replaced with 250 *μ*L of medium or serum-free medium, supplemented with TNF*α* (10 ng/ml) or IL-1*β* (10 ng/ml) (PeproTech EC, Ltd., London, UK).

After different lengths of time (6, 24, or 48 hours) at 37°C, the supernatants from two wells for each different treatment and time were pooled in a single tube and frozen at −20°C to be subsequently analyzed for the presence of cytokines, chemokines, and growth factors. The cell monolayers were then washed twice with PBS, Ca^2+^, and magnesium-free medium, dried, and stored at −20°C for the analysis of DNA content.

For the stimulation of keratinocytes from skin biopsies, 8–10 × 10^4^ cells/cm^2^ in 3 ml were seeded in 6-well plate and cultured in low Ca^2+^ medium, with medium changes every two days. When the cells reached 60%–70% confluence, the medium was removed and replaced with 3 ml of serum-free medium, supplemented with TNF*α* (10 ng/ml). After 48 hours of incubation at 37°C, the supernatant was collected, frozen at −80°C, and subsequently analyzed for the presence of cytokines, chemokines, and growth factor as described in the multiplex system section.

### 2.5. Release of Inflammatory Mediators

#### 2.5.1. ELISA

The release of CXCL8/IL8 and VEGF from HaCaT cells was quantified by using two different high sensitivity human ELISA set (Peprotech, Rocky Hill, NJ, USA) following the method described below. Briefly, Corning 96-well EIA/RIA plates from Sigma-Aldrich (Milan, Italy) were coated with the antibodies provided, overnight at 4°C. Matrix metalloproteinase-9 (MMP-9) secretion from HaCaT cells was evaluated by a different ELISA set (RayBio® Human MMP-9 ELISA kit, Norcross, GA) using a precoated 96-well plate, supplied with the kit. In all the three cases, 300 *μ*l of samples was transferred in duplicate into wells at room temperature for 2 hours. The results were detected by spectroscopy (signal read 450 nm, 0.1 s, by VictorTM X3) using biotinylated and streptavidin-HRP conjugate antibodies, evaluating 3,5,3,59-tetramethylbenzidine (TMB) substrate reaction. The quantification of analytes was done using an optimized standard curve supplied with the ELISA sets. The data are expressed as pg/10^6^ cells. Results are mean ± SD of at least three independent cell culture experiments in duplicate.

#### 2.5.2. Bioplex Multiplex System

Supernatants obtained from HaCaT cells and from primary keratinocytes, stimulated or not with TNF*α* (10 ng/ml) for 48 hours, were analyzed for the presence of chemokines, cytokines, and growth factors. Regarding chemokines, we assayed CXC chemokine ligands (CXCL1/GRO, CXCL10/IP10, CXCL12/SDF-1, and CX3CL1/fractalkine) and CC chemokine ligands (CCL2/MCP1, CCL3/MIP1a, CCL4/MIP1b, CCL5/RANTES, CCL7/MCP3, CCL11/eotaxin, and CCL22/MDC). Among cytokines, we assayed TGF*α*, TNF*β*, IFN-*α*2, IFN-*γ*, G-CSF, GM-CSF, IL-10, IL-12p70, IL-15, and IL-33. All factors were quantified simultaneously by Bio-Plex Pro human cytokine assays according to the manufacturer's instructions (Bio-Plex Bio-Rad Laboratories, CA, USA). The analyte levels were determined using Bio-Plex array reader (Luminex, Austin, TX, USA) and the Bio-Plex manager software. The relative concentration of each analyte was obtained through the establishment of standard curves, and results are expressed as pg/10^6^ cells.

### 2.6. Analysis of DNA Content

Analysis of DNA content was performed using the commercial kit fluorimetric “FluoReporter Blue Fluorometric DNA Quantitation Kit” (Molecular Probes, Life Technologies, Carlsbad, CA, USA) in 96-well plates following the manufacturer's instructions. Plates with treated HaCaT cells were thawed, and 200 *μ*L of distilled H_2_O was added to each well. After three cycles of freezing-thawing, 100 *μ*l was then transferred into a 96-well plate and used for the assay. In parallel, the standard calibration curve was made using increasing concentrations (0–1000 ng/100 *μ*l) of DNA from calf thymus diluted in TE buffer (10 mM Tris base, 1 mM EDTA in distilled H_2_O entire solution with a pH equal to 7.4). The amount of 100 *μ*L of Hoechst dye was then added to each well containing the sample or standard, previously diluted 1 : 400 in TNE buffer (10 mM Tris, 2 M NaCl, 1 mM EDTA with a final pH of 7.4, and 2 mM sodium azide). The emitted fluorescence was measured with microplate reader TECAN F500 (Tecan, Maennedorf, Switzerland) using excitation and emission wavelengths at 346 nm and 460 nm, respectively. The DNA content was calculated by interpolation of the absorbance of the samples on the calibration curve. The DNA content was expressed as *μ*g DNA/10^6^ cells counted, in each well, using a Burker chamber and trypan blue.

### 2.7. Protein Extraction and Analysis of Protein Content

For total protein extraction, HaCaT cells were cultured for 6 or 14 days with proper medium. For each time-point of the differentiation, cells were washed with cold PBS and lysed in RIPA lysis buffer (0.5% deoxycholate, 1% Nonidet P-40, 0.1% SDS, 100 μg/ml of phenylmethylsulfonyl fluoride (PMSF), 1 mM Na_2_VO_4_, and 8.5 *μ*g⁄ml of aprotinin, in PBS), shaking for 20 min at 4°C. Samples were collected by scraper, incubated for 60 min at 4°C, and centrifuged at 12.000 rpm for 15 min at 4°C, and the supernatant was collected and frozen at −20°C until use. The soluble proteins in the extract were quantified according to the method described by Lowry et al. [19].

### 2.8. Western Blot Analysis

For Western blot analysis, 40 *μ*l of total protein was separated on 7.5% SDS-PAGE gel and transferred to a polyvinylidenedifluoride transfer membrane (PVDF) (Bio-Rad, Richmond, CA, USA) for 16 h at 150 mA, using transfer buffer (25 mM TrisHCl, 190 mM glycine, 20% methanol, and 0.05% SDS). The membranes were blocked by incubation in blocking buffer (PBS containing 0.1% Tween and 5% dried nonfat milk) for 2 h and 30 min at room temperature. Then, membranes were blotted overnight at 4°C with various dilutions of primary antibodies, specifically, rabbit polyclonal IgG anti-involucrin (1 : 2500; Genetex, Irvine, CA, USA), mouse polyclonal IgG anti-cytokeratin 14 (1 : 1500; Santa Cruz Biotechnology Inc., Santa Cruz, CA, USA), and rabbit monoclonal IgG anti-cytokeratin 10 (1 : 10,000; Genetex, Irvine, CA USA). Mouse monoclonal anti-*β*-actin antibody (1 : 6000; Sigma-Aldrich, St. Louis, MO, USA) was used to normalize gel loading. Blots were washed six times with PBS-0.1% Tween and incubated for 1 h at room temperature with horseradish peroxidase-linked secondary antibodies. Involucrin and K10 were detected with a donkey anti-rabbit IgG (1 : 10,000; Santa Cruz Biotechnology Inc., Santa Cruz, CA, USA). For K14 and actin, a goat anti-mouse IgG (1 : 10,000; Santa Cruz Biotechnology Inc., Santa Cruz, CA, USA) was used. All blots were developed by ECL Western blotting detection LiteAblot® plus Kit Reagent (Euroclone S.P.A., Milan, Italy), following the manufacturer's protocol. Immunoreactive proteins were visualized by autoradiography on Hyperfilm ECL (GE Healthcare Life Sciences, UK). The relative intensities of band signals were quantified by digital scanning densitometry, and *β*-actin was used to normalize the results to protein content.

### 2.9. Immunofluorescence

HaCaT and primary keratinocytes, cultured on 8-well slide chambers, were washed with PBS, fixed, and permeabilized in ice-cold methanol for 5 min. First, cells were incubated in PBS containing 0.1% bovine serum albumin (BSA) for 10 min, then were incubated with primary antibodies against K10 (rabbit monoclonal IgG anti-cytokeratin 10; 1 : 200; Genetex, Irvine, CA, USA) for 60 min in PBS containing 1% BSA. The cells were subsequently incubated with secondary antibody Alexa Fluor® 488-labeled goat anti-rabbit IgG and Alexa Fluor 488-labeled goat anti-mouse IgG (1 : 1000, Invitrogen, Life Technologies, Carlsbad, CA, USA) for 30 min, respectively. The nuclei of the cells were stained with 4′,6-diamidino-2-phenylindole (DAPI; Sigma-Aldrich, St. Louis, MO, USA), which specifically recognizes DNA. The slides were observed by using Nikon Eclipse TE200 inverted microscope with immersion objective at 60x magnification and photographed with Nikon digital camera (Nikon, Japan).

### 2.10. Statistical Analysis

Data are expressed as mean ± SD of at least three experiments performed in duplicate. Data were analysed by unpaired one-way analysis of variance (ANOVA) followed by Bonferroni post hoc test. Statistical analysis was done using GraphPad Prism 5.0 software (GraphPad Software Inc., San Diego, CA, USA). *p* < 0.05 was considered statistically significant.

## 3. Results

### 3.1. Effect of Extracellular Ca^2+^ and Cell Density on Proliferation and Differentiation of HaCaT Cells

To set up and validate HaCaT cells as an *in vitro* model to study the inflammatory response of human keratinocytes, two known stimuli of KC differentiation, cell density and extracellular Ca^2+^ concentration, were used. HaCaT cells were plated at the same density in low (0.07 mM) and high (1.8 mM) Ca^2+^ medium, and cell proliferation was assessed by both MTT assay and cell counts at day 6 and day 14.

**Figure 2:**
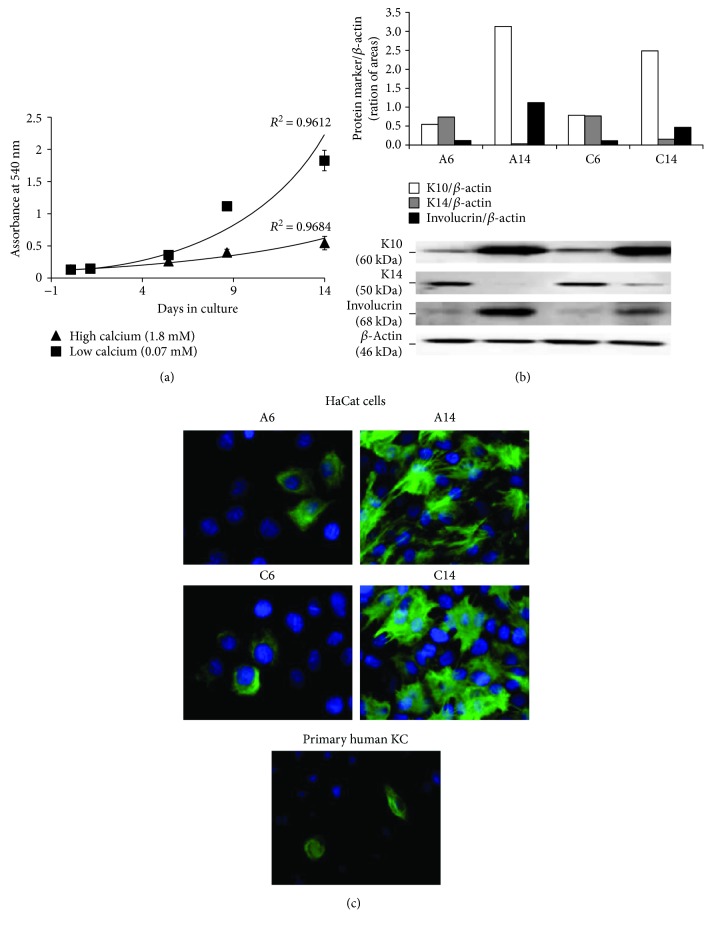
Proliferation and differentiation of HaCaT cells in low (0.07 mM) and high (1.8 mM) Ca^2+^-containing medium. (a) Proliferation of HaCaT cells plated at the same density (1.0 × 10^4^ cells/cm^2^) assessed by the MTT assay at 2, 6, 9, and 14 days of incubation. Values represent mean ± SD of three independent experiments. (b) Western blot analysis of the expression of keratinocyte (KC) differentiation markers (K10, K14, and involucrin) in HaCaT cells grown in low (a) and high (c) Ca^2+^-containing medium for 6 (A6 and C6) or 14 (A14 and C14) days. The relative intensities of band signals quantified by digital scanning densitometry are reported in the histogram; *β*-actin was used to normalize the results to protein content. This blot is a representative of three independent experiments. (c) Immunofluorescence staining of HaCaT cells, grown in low (A) and high (C) Ca^2+^-containing medium for 6 (A6 and C6) and 14 (A14 and C14) days, and of primary human KC, grown in low Ca^2+^-containing medium, with anti-K10 antibodies (magnification: 60x).

In low Ca^2+^ medium, a steady increase in metabolic activity and cell number was observed over time, while in high Ca^2+^ medium, cell growth was slower. Comparing low to high Ca^2+^ medium, about 27.5% and 70.3% decrease of MTT activity and 40% and 60% decrease in cell count were seen at days 6 and 14, respectively (Figure 2(a) and Table 1S). However, no significant changes in cell morphology were observed in the different conditions. Phase contrast images showed that HaCaT cells were flat and spread out after 6 days of growth both in low (A6) or high (C6) Ca^2+^ medium when they were at 80% confluence; at day 14 both in low (A14) and high (C14) Ca^2+^ concentration, they became more cubical in shape with higher cell-to-cell packing and stratification (Figure S1). As reported in Table 1S, no significant changes were observed in the DNA and protein content in HaCaT cells grown in medium with different Ca^2+^ concentrations at the 6th or 14th day.

The expression of three classical KC differentiation markers (K14, K10, and involucrin) in response to changes in cell density and extracellular Ca^2+^ levels was assessed by Western blot analysis. Physiologically, the expression of K14 is restricted to the epidermal basal layer, while the presence of K10 and involucrin indicates a more differentiated phenotype.

As shown in Figure 2(b), the expression of K14 was higher in HaCaT cells at 80% confluence (day 6) than in overconfluent cells (day 14). At the same time, the levels of K10 and involucrin increased from day 6 to day 14, confirming the role of cell density on differentiation. Comparing day 6 to day 14, the fold increase in low Ca^2+^ medium was more evident for involucrin than for K10 (10.4-fold versus 5.7-fold, resp.), whereas in high Ca^2+^ medium, the increase was 4.7-fold versus 3.2-fold, respectively.

Regarding the effect of Ca^2+^, at day 6, HaCaT cells showed similar levels of K14, K10, or involucrin in A6 and C6 culture conditions, suggesting that Ca^2+^ concentration did not significantly influence their expression at 80% cell confluence (Figure 2(b)). Unexpectedly, at day 14, the levels of both K10 and involucrin were slightly lower in overconfluent HaCaT cells grown in high compared to low Ca^2+^ medium (Figure 2(b)). This result could be a consequence of their lower density (Table 1S).

The acquisition of HaCat cell-differentiated phenotype was confirmed by immunofluorescence experiments (Figure 2(c)). The expression of the differentiation marker K10 increased significantly in HaCaT cells at day 14 compared to day 6 with almost 100% cells being positive. In addition, again, no differences in K10 levels were seen between HaCaT cells grown in low or high Ca^2+^ medium both in 80% confluent or overconfluent cells. Control samples without primary antibodies were negative, confirming specificity (data not shown).

The pattern of K10 expression was also evaluated on primary human keratinocytes grown in low Ca^2+^ and serum-free medium. Interestingly, as shown in Figure 2(c), K10 expression on human primary keratinocytes paralleled HaCaT cells grown for 6 days in low Ca^2+^ medium (A6).

### 3.2. Release of Inflammatory Mediators from HaCaT Cells

HaCaT cells grown in different culture conditions described above were then utilized to evaluate the production of a series of bioactive molecules, known to be released in the skin during inflammation or repair, both in basal conditions and upon proinflammatory stimulation.

In a first set of experiments, we focused on three main mediators: CXCL8/IL8, VEGF, and MMP-9, which are crucial for inflammatory cell recruitment, angiogenesis, and matrix remodeling, respectively. TNF*α* and IL-1*β* were used as stimuli. Influence of serum in the medium was also verified.

**Figure 3:**
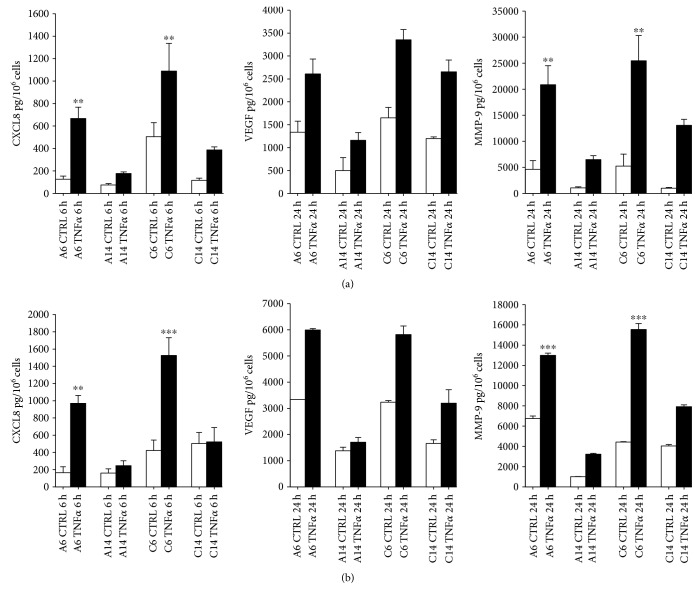
*In vitro* release of CXCL8/IL8, VEGF, and MMP-9 from HaCaT cells stimulated by TNF*α* during cell differentiation. The amount of CXCL8/IL8, VEGF, and MMP-9 was measured by ELISA in the supernatants of HaCaT cells plated at the same density (1.0 × 10^4^ cells/cm^2^), grown in low (a) and high (C) Ca^2+^-containing medium for 6 (A6 and C6) or 14 (A14 and C14) days (white bars), and treated with 10 ng/ml TNF*α* for 6 or 24 hours (black bars), as indicated, in the absence (a) or the presence (b) of serum. The data are expressed as pg/10^6^ cells, and values are the mean ± SD of at least three independent experiments in duplicate.

The supernatants were obtained from HaCaT cells plated at the same density in low or high Ca^2+^ medium and treated, after 6 or 14 days of culture, with 10 ng/ml TNF*α* or IL-1*β* for 6 or 24 h, as indicated, in absence (Figure 2(a)) or presence of serum (Figure 2(b)). The supernatants were recovered to measure CXCL8/IL8, VEGF, and MMP-9 by ELISA tests. The results are reported in Figure 3 and Figure S2.

Compared to basal levels, the stimulation with TNF*α* in the absence of serum induced a significant increase of CXCL8/IL8 and MMP-9, but not of VEGF, both in low and high Ca^2+^ medium at day 6 (Figure 3(a)). Conversely, the presence of serum during TNF*α* treatment at day 6 that did not elicit appreciable effects on CXCL8/IL8 release lowered the TNF*α*-induced MMP-9 release and increased both basal and stimulated levels of VEGF (Figure 3(b)). The concentration fold increase, following TNF*α* stimulation, was serum independent.

Overall, it appears that the ability of HaCaT cells of releasing these mediators decreased appreciably at day 14, compared to day 6, and this is independent on the Ca^2+^ concentration or the presence of serum (Figure 3). Of note, the MMP-9 amount released at day 6 and day 14 is higher in the absence than in the presence of serum, both in basal condition and upon stimulation.

Similar profiles were observed when cells were stimulated with IL-1*β* (10 ng/ml) (Figure S2). Higher levels of CXCL8/IL8, VEGF, and MMP-9 were seen at day 6, compared to day 14, independently of serum or Ca^2+^ concentration. In the presence of serum, unstimulated cells produced higher levels of mediators than without serum, and, except for IL8, the increase of VEGF or MMP-9 triggered by IL-1*β* was not significant.

**Figure 4:**
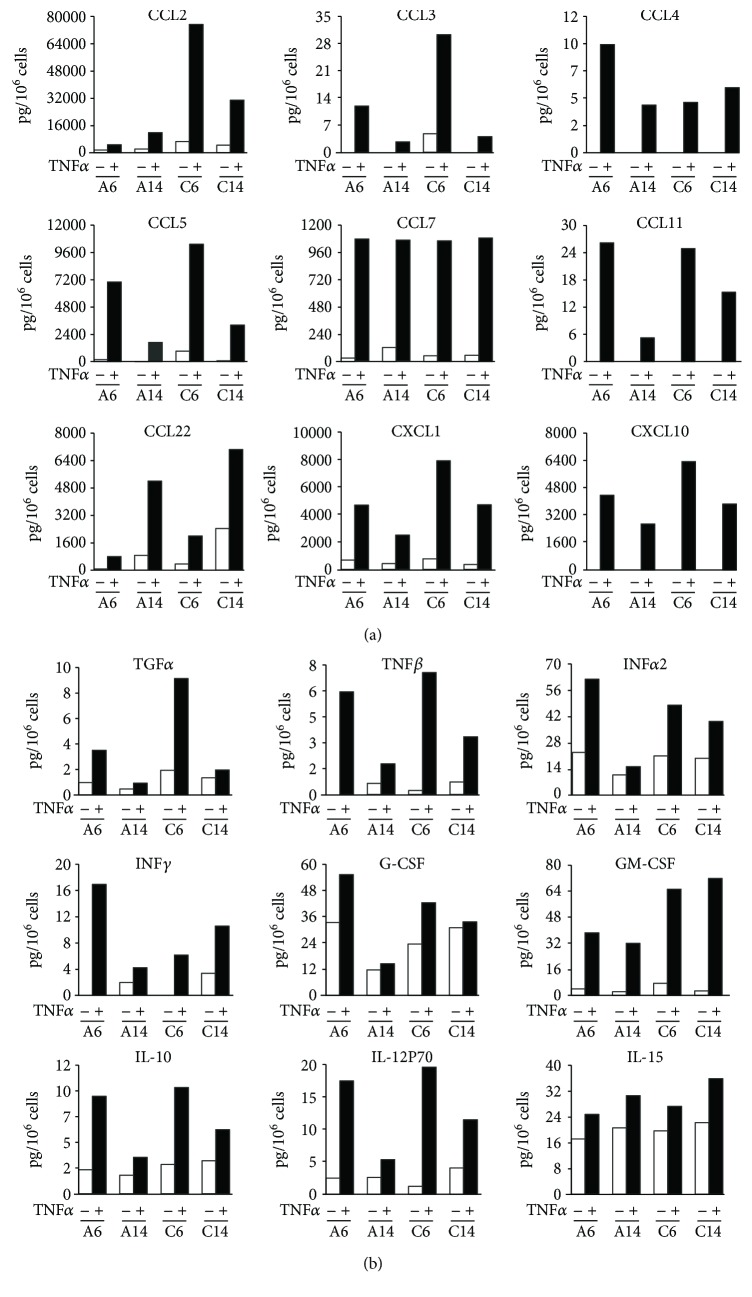
Bioplex multiplex system analysis of supernatants from HaCaT cells stimulated by TNF*α* during cell differentiation. The amount of chemokines (a), cytokines, and growth factors (b) was measured by Multiplex system technology in the supernatants of HaCaT cells plated at 1.0 × 10^4^ cells/cm^2^, grown in low (a) and high (c) Ca^2+^-containing medium for 6 (A6 and C6) and 14 (A14 and C14) days (white bars), and treated with 10 ng/ml TNF*α* for 48 hours (black bars) in a serum-free medium. The data are expressed as pg/10^6^ cells.

To extend these observations, different chemokines and cytokines were studied by magnetic bead suspension array using the Bio-Plex Pro technology. The experiments were conducted in serum-free medium to reduce the interferences described in the previous paragraph. As shown in Figures 4(a) and 4(b), the stimulation of HaCaT cells with TNF*α* (10 ng/ml) for 48 h resulted in the upregulation of secretion of almost all the 18 mediators tested in the different culture conditions, although the level of stimulation varied among them. In particular, 6 days of culture in high Ca^2+^ concentration seem to represent the best combination for optimal production of the majority of mediators, except for CCL4/MIP1b, INF*α*2, INF*γ*, and G-CSF. Of note, CCL7/MCP3 seemed to be the only cytokine strongly upregulated by TNF*α* independently of the day of culture and Ca^2+^ concentration, whereas CCL22/MDC was the only cytokine for which overconfluency, and not Ca^2+^ levels, contributed to its production. On the contrary, Ca^2+^ concentration, but not the days in culture, seemed to be particularly relevant for TNF*α*-induced release of GM-CSF. No measurable values for CXCL12/SDF-1, CX3CL1/fractalkine, or IL-33 were obtained from any samples (data not shown).

### 3.3. Release of Inflammatory Mediators from Primary Human Keratinocytes

**Figure 5:**
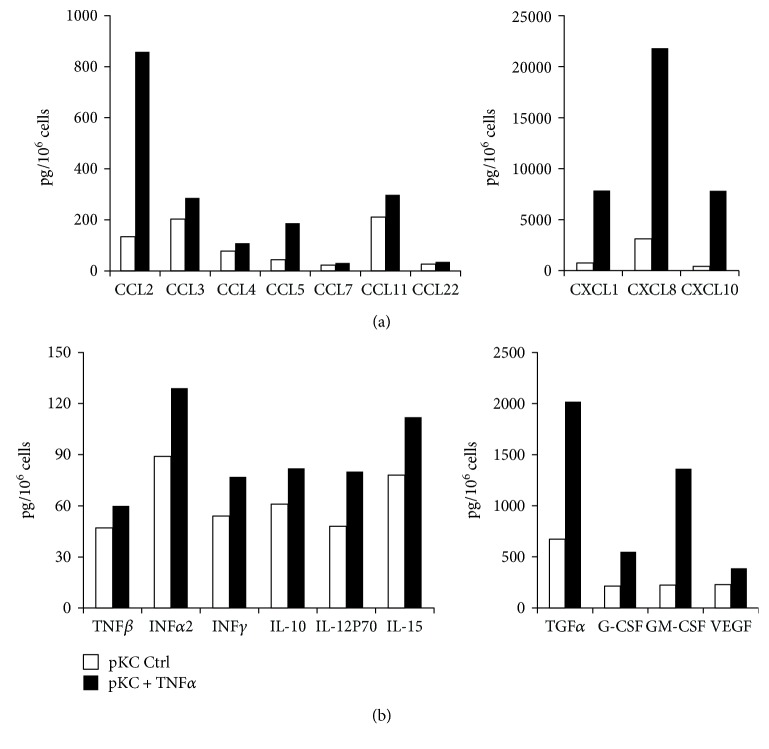
*In vitro* release of cytokines, chemokines, and growth factors from primary adult human keratinocytes stimulated by TNF*α*. The amount of chemokines (a), of cytokines, and of growth factors (b) was measured by Multiplex system technology in the supernatants of primary adult human keratinocytes (pKC) plated at the same density (8–10 × 10^4^ cells/cm^2^) and grown in low Ca^2+^-containing medium (white bars). The treatment with 10 ng/ml TNF*α* was carried out for 48 hours in a serum-free medium when cells were 60–70% confluent (black bars). The data are expressed as pg/10^6^ cells.

The release of inflammatory mediators was also measured in primary nonlesional epidermal KCs obtained from psoriatic patients. These cells were grown in low Ca^2+^ medium and, when tested at 60–70% confluence, showed K10 expression comparable to A6 HaCaT cells (Figure 2(c)). Regarding the production of cytokines/chemokines, normal KCs secreted basal level of all the molecules analyzed and the treatment with TNF*α* induced a further increase of almost all of them (Figures 5(a) and 5(b)). All the CXC chemokine family members appeared to be highly upregulated, reaching a value for the CXCL8/IL8 more than 20,000 pg/10^6^ cells (Figure 5(a)). Among the CC chemokines, only CCL2 showed approximately fivefold increase above controls. Of note, high concentration of growth factors, as TGF*α* and GM-CSF, was detected in medium of primary KCs stimulated with TNF*α* (Figure 5(b)).

## 4. Discussion

Keratinocytes are active players in epidermal repair and in the skin's immune defense through the secretion of growth factors, cytokines, and chemokines. To facilitate and standardize the *in vitro* studies on KCs, we investigated the use of HaCaT cells as a suitable model to follow the release of cutaneous inflammatory and repair mediators in response to TNF*α* or IL-1*β*, and in relation to different culture conditions and differentiation levels.

HaCaT cells are a long-lived, spontaneously immortalized human KC line, which exhibit basal cell properties and display substantial changes in response to two well-established *in vitro* prodifferentiating agents: the increase in cell density, as a function of time in culture, and extracellular Ca^2+^ concentration.

The switch from low to high extracellular Ca^2+^ concentration is considered not only a major regulator of the KC differentiation, but also of their proliferation both *in vitro* and *in vivo*. A Ca^2+^ gradient within the epidermis promotes the sequential differentiation of KC from the basal layer to the stratum corneum. Moreover, Ca^2+^ regulates the formation of desmosomes, adherent junctions, and tight junctions. The latter maintain cell-cell adhesion and play an important role in intracellular signaling that regulate Ca^2+^ levels and cell cycle arrest, critical for the differentiation process [12].

With this in mind, we first tested the differentiation potential of HaCaT cells in response to the increase in cell density and in extracellular Ca^2+^ concentration, by evaluating the expression of three markers of differentiation, namely K14, whose expression is restricted to the basal layer, K10, and involucrin that indicate a more differentiated phenotype. Our data (Figure 1 and Table 1S) confirm and extend previous observations that the increase in cell density and extracellular Ca^2+^ concentration favors HaCaT differentiation [14–17]. HaCaT cells remain in the basal state (high levels of K14) when maintained in low Ca^2+^ conditions and less than 80% confluent (A6), while they begin to differentiate expressing high levels of K10 and involucrin after a long-term culture (A14), that is when, although maintained in low Ca^2+^ medium, they are overconfluent. Different from previous observations, we noticed that HaCaT cells maintain their basal phenotype also when the cells are cultured in high Ca^2+^ conditions, but at less than 80% confluence. This observation suggests that, although extracellular Ca^2+^ > 0.1 mM appears to be a major regulator of HaCaT differentiation, the role of cell density is also relevant and it might have been underestimated in previous *in vitro* studies.

Moreover, in our study, the Western blot analysis of K10 and involucrin expression showed that when HaCaT cells are maintained in high Ca^2+^ condition, the overconfluence promotes a slight decrease of both K10 and involucrin levels and a weak increase of K14. Although this observation may be a consequence of the lower density of C14 with respect to A14, these data are in agreement with the changes of Ca^2+^ and confluence-dependent expression of K1, a biomarker of more differentiated cells [20], but not with the changes reported for the transglutaminase biomarker [17]. Altogether, this emphasizes the importance of a careful monitoring of differentiation marker expression during long-term culture of HaCaT cells and of standardization of the culture conditions.

The effect of the Ca^2+^ switch on the proliferation of HaCaT cells is poorly investigated. Our viability assays and cell counts highlight that the extracellular Ca^2+^ concentration strongly influences the proliferative ability of HaCaT cells. Up to day 2 of culture, no differences in the proliferation rate of HaCaT cells grown in low or high Ca^2+^ medium were seen, whereas a difference in the growth rate was evident in low Ca^2+^ medium starting from day 6, which became significantly higher at day 14. These findings are at variance of those showing a progressive, time-dependent increase of HaCaT proliferation in high Ca^2+^ medium [14]. A possible explanation could be the higher extracellular Ca^2+^ concentration used in the present study (1.8 mM) compared to 1.2 mM in [14]. In fact, it has been demonstrated that only an increase in intracellular Ca^2+^ concentration above 1.5 mM results in a reduced growth rate [21].

In the second part of this study, HaCaT cells, cultured in low or high Ca^2+^ medium for different lengths of time, were used to investigate the release of CXCL8/IL8, VEGF, and MMP-9 in response to two proinflammatory stimuli, TNF*α*, and IL-1*β*. These cytokines were chosen for their predominant role in the pathogenesis of skin inflammation, since they both regulate genes previously shown to be specifically overexpressed in psoriasis [10, 22–24]. Our results show that the release of CXCL8/IL8, VEGF, and MMP-9 was higher in HaCaT cell basal state (A6-C6) compared to more differentiated cells (A14-C14). Ca^2+^ concentrations slightly enhanced the secretion of these mediators, especially when IL-1*β* was used as proinflammatory stimulus; this effect could be partially explained by the activation of the Ca^2+^ responsive promoter of activator protein (AP)-1 [25].

The presence of serum reduced the TNF*α*-induced release of MMP-9 and enhanced the VEGF secretion, both at basal and stimulated levels. As previously reported, growth factors derived from fetal calf serum may influence a variety of parameters involved in KC proliferation, differentiation, and wound healing processes [26–28]. Based on these results, we strongly suggest that serum should not be used when the proinflammatory mediators, VEGF, or MMP-9, are assayed. This should be extended also to the assays using normal human KC.

Several papers demonstrated that TNF*α* in KC regulates different genes involved in inflammation and angiogenesis, such as IL-1, ICAM-1, VEGF [29, 30], TGF-*β*, chemokines (CCL20, CCL27, CCL5, CCL2, CXCL10, and CXCL11), and members of the CXCL8 family, including CXCL1, CXCL2, and CXCL3 [10, 22, 23]. Therefore, using the Multiplex technology, we evaluated the effect of Ca^2+^ concentration and cellular density on the secretion of different cytokines, chemokines, and growth factors in HaCaT cell line. All the analyzed parameters were upregulated to a great extent by TNF*α* at day 6 in comparison to day 14; only CCL22/MDC seemed to be preferentially released in more differentiated HaCaT cells. CCL4, INF*α*2, INF*γ*, and G-CSF had a higher release in low Ca^2+^ condition, whereas, on the opposite, in high Ca^2+^ concentration, a comparable or higher secretion of CCL2, CCL3, CCL5, CCL7, CCL11, CCL22, CXCL1, CXCL10, TGF*α*, TNF-*β*, IL-10, and IL-15 was seen. To summarize, both long-term culture and Ca^2+^ concentration in the medium affect the ability of HaCaT cells to release chemokines and growth factors.

Finally, to validate HaCaT cells as a reliable model to dissect the inflammatory/repair response of human KCs, we measured the release of inflammatory mediators in primary nonlesional epidermal KCs obtained from psoriatic patients. These cells were assayed in low Ca^2+^medium and in the absence of serum.

The levels of the CXC family of chemokines, induced by TNF*α* on human KCs (CXCL1, CXCL8, and CXCL10) correlated relatively well to the values released by HaCaT cells in low Ca^2+^, considering the fold induction. Similar results were obtained on growth factors released (TGF*α*, G-CSF, GM-CSF, and VEGF). Not so close was the correlation with the CC family of chemokines some of which (CCL4, CCL5, CCL7, and CCL11) were highly released in A6 and C6 HaCaT cells, but quite low in normal human KCs; CCL2 was higher in C6 HaCaT as in normal KCs, whereas CCL3 was similar. Our results also show that HaCaT cells especially at day 14 of culture produce more CCL22/MDC than normal human KCs, thus confirming previously published data [31, 32]. This is not the first time that a differential release of chemokines is described in primary human KCs or HaCaT cells stimulated with exogenous stimuli. Data in the literature reported that CXCL10 and IL8 were released to a similar extent by both cell types, while CXCL9 and CCL20 were more efficiently produced by primary human KCs [33].

In conclusion, this study is the first report where several variables, all together, including the influence of Ca^2+^ concentration, the cell density, the differentiation state, and the presence of serum, were considered as factors that may influence release of proinflammatory mediators by KCs. Our results support the use of HaCaT cell line, under carefully optimized *in vitro* condition, as a reliable model, with respect to normal KCs, to screen for new anti-inflammatory compounds for skin diseases. Indeed, HaCaT cell line has been successfully used for studying those pathologies in which skin keratinocytes are involved, such as infectious diseases or tumors, or as *in vitro* carcinogenesis model of human skin [34]. By modulating Ca^2+^ concentration in culture medium and maintaining 80% confluence, HaCaT cells are in the conditions of producing cytokines at medium/low levels (A6 condition) or at medium/high levels (C6 condition) as expected in highly activated KCs from skin lesions. This *in vitro* system has the advantage of being reproducible and reliable and definitively less invasive and with less variability than KC from skin biopsies. However, one of the drawbacks is the inability of reproducing the skin complexity and cellular heterogeneity in basal or inflammatory conditions. 2D-3D culture systems have been proposed which mimic the differentiation process [35]. It cannot be excluded that in a relatively short time we may have available 3D culture systems in which several cell lineages KC, immune cells, and fibroblasts will be cocultured to ensure a better reproduction of the skin microenvironment.

## Abbreviations

CCL: C-C motif chemokine ligand
CXCL: C-X-C motif chemokine ligand
CX3CL: C-X3-C motif chemokine ligand
G-CSF: Granulocyte-colony stimulating factor
GM-CSF: Granulocyte-macrophage colony-stimulating factor
IFN: Interferon
IL: Interleukin
K: Keratin
KC: Keratinocyte
MMP-9: Matrix metalloproteinase-9
TGF*α:*: Transforming growth factor alpha
TNF*α:*: Tumor necrosis factor alpha
TNF*β:*: Tumor necrosis factor beta
VEGF: Vascular endothelial growth factor.

## Supplementary Materials

Supplementary 1 Table 1S: Cell recovery and protein content of HaCaT cells grown in low (A) or high (C) Ca^2+^ medium for 6 (A6, C6) or 14 (A14, C14) days.

Supplementary 2 Figure S1: Changes in HaCaT cell morphology during cell differentiation.

Supplementary 3 Figure S2: In vitro release of CXCL8/IL8, VEGF, and MMP-9 from HaCat cells stimulated by IL-1*β* during cell differentiation.

## References

[1] Baroni A., Buommino E., De Gregorio V., Ruocco E., Ruocco V., Wolf R. (2012). Structure and function of the epidermis related to barrier properties. *Clinics in Dermatology*.

[2] Hanel K. H., Cornelissen C., Luscher B., Baron J. M. (2013). Cytokines and the skin barrier. *International Journal of Molecular Sciences*.

[3] Henry J., Toulza E., Hsu C. Y. (2012). Update on the epidermal differentiation complex. *Frontiers in Bioscience*.

[4] Proksch E., Brandner J. M., Jensen J. M. (2008). The skin: an indispensable barrier. *Experimental Dermatology*.

[5] Nestle F. O., Di Meglio P., Qin J. Z., Nickoloff B. J. (2009). Skin immune sentinels in health and disease. *Nature Reviews Immunology*.

[6] Suter M. M., Schulze K., Bergman W., Welle M., Roosje P., MÃ¼ller E. J. (2009). The keratinocyte in epidermal renewal and defence. *Veterinary Dermatology*.

[7] Albanesi C., De Pita O., Girolomoni G. (2007). Resident skin cells in psoriasis: a special look at the pathogenetic functions of keratinocytes. *Clinics in Dermatology*.

[8] Pastore S., Mascia F., Girolomoni G. (2006). The contribution of keratinocytes to the pathogenesis of atopic dermatitis. *European Journal of Dermatology*.

[9] Albanesi C. (2009). The role of keratinocytes in inflammatory skin diseases. *Advances in Psoriasis and Inflammatory Skin Diseases*.

[10] Albanesi C., Scarponi C., Giustizieri M. L., Girolomoni G. (2005). Keratinocytes in inflammatory skin diseases. *Current Drug Target -Inflammation & Allergy*.

[11] Bikle D. D., Ratnam A., Mauro T., Harris J., Pillai S. (1996). Changes in calcium responsiveness and handling during keratinocyte differentiation. Potential role of the calcium receptor. *The Journal of Clinical Investigation*.

[12] Bikle D. D., Xie Z., Tu C. L. (2012). Calcium regulation of keratinocyte differentiation. *Expert Review of Endocrinology & Metabolism*.

[13] Schurer N., Kohne A., Schliep V., Barlag K., Goerz G. (1993). Lipid composition and synthesis of HaCaT cells, an immortalized human keratinocyte line, in comparison with normal human adult keratinocytes. *Experimental Dermatology*.

[14] Micallef L., Belaubre F., Pinon A. (2009). Effects of extracellular calcium on the growth-differentiation switch in immortalized keratinocyte HaCaT cells compared with normal human keratinocytes. *Experimental Dermatology*.

[15] Boukamp P., Petrussevska R. T., Breitkreutz D., Hornung J., Markham A., Fusenig N. E. (1988). Normal keratinization in a spontaneously immortalized aneuploid human keratinocyte cell line. *The Journal of Cell Biology*.

[16] Deyrieux A. F., Wilson V. G. (2007). In vitro culture conditions to study keratinocyte differentiation using the HaCaT cell line. *Cytotechnology*.

[17] Garach-Jehoshua O., Ravid A., Liberman U. A., Reichrath J., Glaser T., Koren R. (1998). Upregulation of the calcium-dependent protease, calpain, during keratinocyte differentiation. *British Journal of Dermatology*.

[18] Mosmann T. (1983). Rapid colorimetric assay for cellular growth and survival: application to proliferation and cytotoxicity assays. *Journal of Immunological Methods*.

[19] Lowry O. H., Rosebrough N. J., Farr A. L., RANDALL R. O. S. E. J. (1951). Protein measurement with the Folin phenol reagent. *Journal of Biological Chemistry*.

[20] Capone A., Visco V., Belleudi F. (2000). Up-modulation of the expression of functional keratinocyte growth factor receptors induced by high cell density in the human keratinocyte HaCaT cell line. *Cell Growth & Differentiation*.

[21] Sakaguchi M., Miyazaki M., Takaishi M. (2003). S100C/A11 is a key mediator of Ca^2+^-induced growth inhibition of human epidermal keratinocytes. *The Journal of Cell Biology*.

[22] Banno T., Gazel A., Blumenberg M. (2004). Effects of tumor necrosis factor-α (TNFα) in epidermal keratinocytes revealed using global transcriptional profiling. *Journal of Biological Chemistry*.

[23] Albanesi C., Pastore S. (2010). Pathobiology of chronic inflammatory skin diseases: interplay between keratinocytes and immune cells as a target for anti-inflammatory drugs. *Current Drug Metabolism*.

[24] Yano S., Banno T., Walsh R., Blumenberg M. (2008). Transcriptional responses of human epidermal keratinocytes to cytokine interleukin-1. *Journal of Cellular Physiology*.

[25] Elsholz F., Harteneck C., Muller W., Friedland K. (2014). Calcium - a central regulator of keratinocyte differentiation in health and disease. *European Journal of Dermatology*.

[26] Altankov G., Hecht J., Dimoudis N. (2001). Serum-free cultured keratinocytes fail to organize fibronectin matrix and possess different distribution of beta-1 integrins. *Experimental Dermatology*.

[27] Hubner G., Werner S. (1996). Serum growth factors and proinflammatory cytokines are potent inducers of activin expression in cultured fibroblasts and keratinocytes. *Experimental Cell Research*.

[28] Pfundt R., Wingens M., Bergers M., Zweers M., Frenken M., Schalkwijk J. (2000). TNF-α and serum induce SKALP/elafin gene expression in human keratinocytes by a p38 MAP kinase-dependent pathway. *Archives of Dermatological Research*.

[29] Barker J. N., Mitra R. S., Griffiths C. E., Dixit V. M., Nickoloff B. J. (1991). Keratinocytes as initiators of inflammation. *The Lancet*.

[30] Yoshida S., Ono M., Shono T. (1997). Involvement of interleukin-8, vascular endothelial growth factor, and basic fibroblast growth factor in tumor necrosis factor alpha-dependent angiogenesis. *Molecular and Cellular Biology*.

[31] Yano C., Saeki H., Komine M. (2015). Mechanism of macrophage-derived chemokine/CCL22 production by HaCaT keratinocytes. *Annals of Dermatology*.

[32] Kobayashi M., Shimauchi T., Hino R., Tokura Y. (2004). Roxithromycin downmodulates Th2 chemokine production by keratinocytes and chemokine receptor expression on Th2 cells: its dual inhibitory effects on the ligands and the receptors. *Cellular Immunology*.

[33] Olaru F., Jensen L. E. (2010). Chemokine expression by human keratinocyte cell lines after activation of toll-like receptors. *Experimental Dermatology*.

[34] Fusenig N. E., Boukamp P. (1998). Multiple stages and genetic alterations in immortalization, malignant transformation, and tumor progression of human skin keratinocytes. *Molecular Carcinogenesis*.

[35] Li L., Fukunaga-Kalabis M., Herlyn M. (2011). The three-dimensional human skin reconstruct model: a tool to study normal skin and melanoma progression. *Journal of Visualized Experiments*.

