# Supplementary material for: HaCaT Cells as a Reliable In Vitro Differentiation Model to Dissect the Inflammatory/Repair Response of Human Keratinocytes
Source: Mediators Inflamm. 2017 Dec 17;2017:7435621. doi: 10.1155/2017/7435621 (PMC5748104; doi:10.1155/2017/7435621)
Supplement: Supplementary 1 — Table 1S: Cell recovery and protein content of HaCaT cells grown in low (A) or high (C) Ca2+ medium for 6 (A6, C6) or 14 (A14, C14) days. [file 7435621.f1.pdf]

Supplementary

Table *1S*: Cell recovery and protein content of HaCaT cells grown in low (A) or high (C) Ca^{2+} medium for 6 (A6, C6) or 14 (A14, C14) days

Cell culture	Cells number<br><br>10^6/well ± s.d.	Protein<br><br>μg/10^6 cells ± s.d.
A6	10.14 ± 1.51[a]	192.39 ± 46.90[c]
C6	6.08 ± 1.66[a]	194.04 ± 51.93[c]
A14	38.27 ± 6.38[b]	132.32 ± 39.13[c]
C14	15.43 ± 5.30[b]	143.40 ± 37.47[c]

Notes:

[a]average from 24 independent cell counts
[b]average from 40 independent cell counts
[c]average from 12 independent assays

Figure legends

Figure *S1*. Changes in HaCaT cell morphology during cell differentiation.

Morphological changes in HaCat cells, grown in low (A) and high (C) Ca^{2+}-containing medium for 6 (A6 and C6) and 14 (A14 and C14) days were analyzed by phase contrast microscopy.

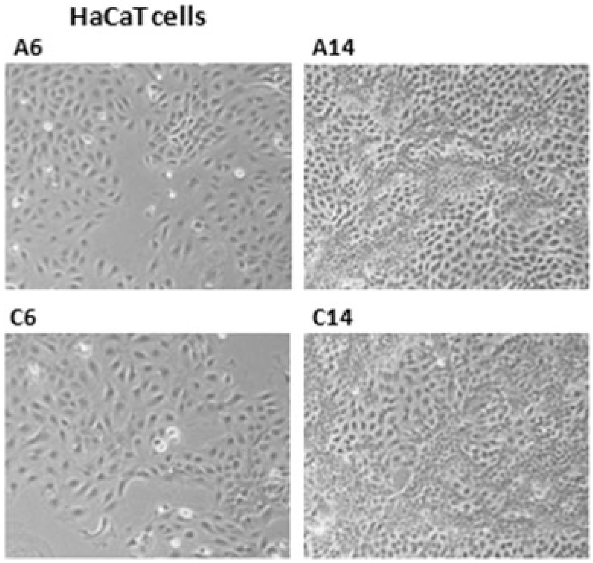

Figure *S2*. *In vitro* release of CXCL8/IL8, VEGF and MMP-9 from HaCat cells stimulated by IL-1β during cell differentiation.

The amount of CXCL8/IL8, VEGF and MMP-9 was measured by ELISA assay in the supernatants of HaCaT cells plated at the same density (1.0×10^4 cells/cm^2), grown in low (A) and high (C) Ca^{2+}-containing medium for 6 (A6 and C6) and 14 (A14 and C14) days (white bars) and treated with 10 ng/ml IL-1β for 6 or 24 hours (black bars), as indicated, in the absence (*Panel A*) or in the presence (*Panel B*) of serum. The data are expressed as pg/10^6 cells and values are the mean ± s.d. of at least three independent cell culture experiments in duplicate.

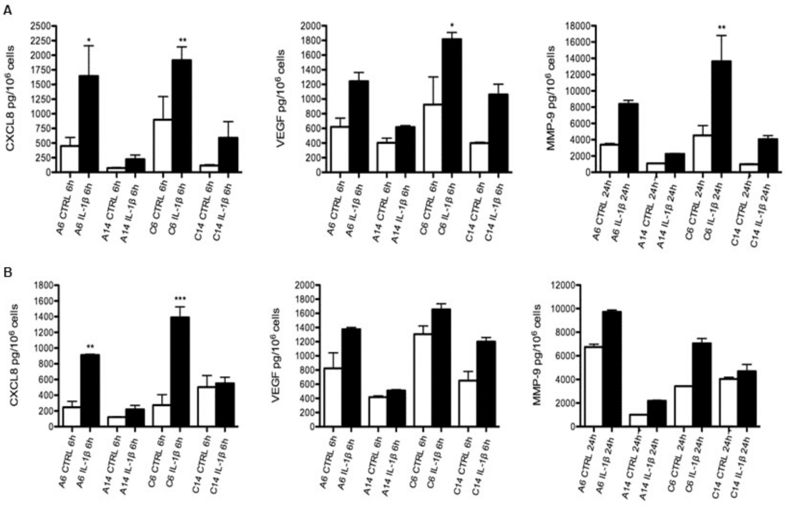
A
B
CXCL8 pg/10^6 cells
VEGF pg/10^6 cells
MMP-9 pg/10^6 cells
A6 CTRL 6h
A6 IL-1β 6h
A14 CTRL 6h
A14 IL-1β 6h
C6 CTRL 6h
C6 IL-1β 6h
C14 CTRL 6h
C14 IL-1β 6h
A6 CTRL 24h
A6 IL-1β 24h
A14 CTRL 24h
A14 IL-1β 24h
C6 CTRL 24h
C6 IL-1β 24h
C14 CTRL 24h
C14 IL-1β 24h
