# Supplementary figures and images for: HaCaT Cells as a Reliable In Vitro Differentiation Model to Dissect the Inflammatory/Repair Response of Human Keratinocytes
Source: Mediators Inflamm. 2017 Dec 17;2017:7435621. doi: 10.1155/2017/7435621 (PMC5748104; doi:10.1155/2017/7435621)

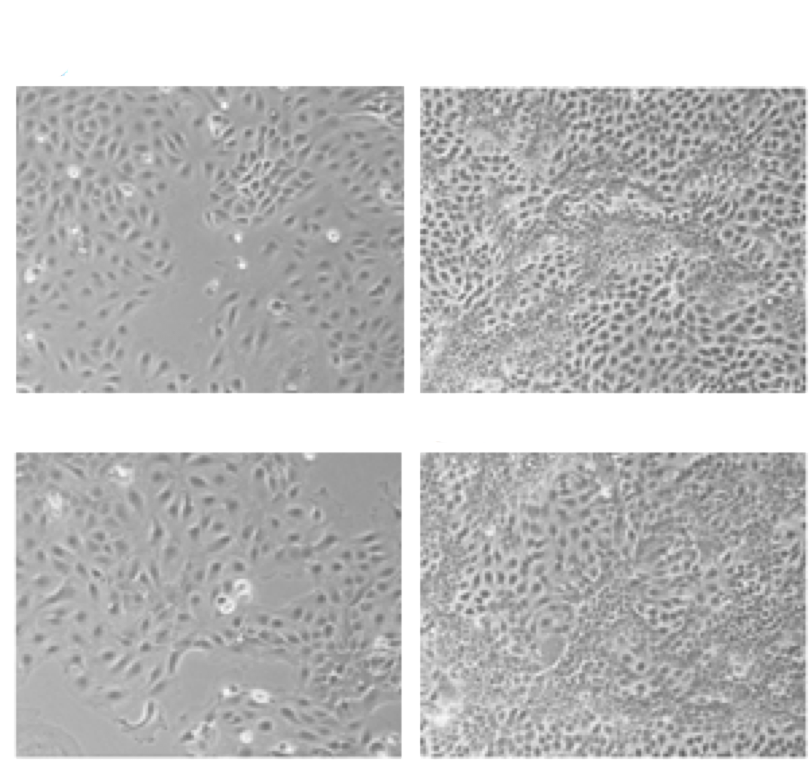


**HaCaT cells**

**C14**

**C6**

**A14**

**A6**

Supplement: Supplementary 2 — Figure S1: Changes in HaCaT cell morphology during cell differentiation. [file 7435621.f2.docx]
